# Prevalence and Risk Factors of Postpartum Depression in Romanian Women during Two Periods of COVID-19 Pandemic

**DOI:** 10.3390/jcm11061628

**Published:** 2022-03-15

**Authors:** Cosmin Citu, Florin Gorun, Andrei Motoc, Ioan Sas, Bogdan Burlea, Ioana Mihaela Citu, Marius Biris, Marius Forga, Octavian Neagoe, Oana Maria Gorun

**Affiliations:** 1 Department of Obstetrics and Gynecology, “Victor Babes” University of Medicine and Pharmacy Timisoara, Eftimie Murgu Square, 300041 Timisoara, Romania; citu.ioan@umft.ro (C.C.); sasioan56@yahoo.com (I.S.); biris.marius@umft.ro (M.B.); forga.marius@umft.ro (M.F.); 2 Department of Anatomy and Embryology, “Victor Babes” University of Medicine and Pharmacy Timisoara, Eftimie Murgu Square, 300041 Timisoara, Romania; amotoc@umft.ro; 3 Department of Obstetrics and Gynecology, Municipal Emergency Clinical Hospital Timisoara, 1–3 Alexandru Odobescu Street, 300202 Timisoara, Romania; bogdanburlea@yahoo.com (B.B.); oanabalan@hotmail.com (O.M.G.); 4 Department of Internal Medicine I, “Victor Babes” University of Medicine and Pharmacy Timisoara, 2 Eftimie Murgu Square, 300041 Timisoara, Romania; citu.ioana@umft.ro; 5 Second Department of General Surgery and Oncology, University of Medicine and Pharmacy Timisoara, Eftimie Murgu Square, 300041 Timisoara, Romania; dr.octavian.neagoe@gmail.com

**Keywords:** postpartum depression, mental health, COVID-19

## Abstract

Postpartum depression is a major mental health disorder that can negatively affect both mother and baby. In addition, the COVID-19 pandemic associated with extreme measures of the lockdown had profound effects on humanity, increasing the rates of anxiety and depression, especially among women in the postpartum period. The aim of this study was threefold: to determine the prevalence of postpartum depression, to compare the prevalence of postpartum depression at two different times during the COVID-19 pandemic, and to assess a possible association between the timing of childbirth in a given period of the pandemic and the risk of postpartum depression. A cross-sectional study involving 154 women who were interviewed immediately postpartum, using the EPDS scale, was conducted at the Timisoara Municipal Hospital, Romania at two different periods during the COVID-19 pandemic (March–April 2020 during the first wave and August–September 2021 during the fourth wave). The overall prevalence of postpartum depression (EPDS score > 13) was 18.8%, with a statistically significantly higher rate among participants surveyed during the fourth wave of the COVID-19 pandemic in Romania; the COVID-19 pandemic represents an impact on women’s mental health in the postpartum period, increasing the risk of developing postpartum depression.

## 1. Introduction

Coronavirus disease 2019 (COVID-19) is a respiratory infectious disease caused by the SARS-CoV-2 virus (severe acute respiratory syndrome coronavirus 2). It was first discovered in Wuhan, China in December 2019, from where it spread fast and caused a global pandemic in a short period of time [1,2,3]. To limit the number of patients infected with COVID-19, governments around the world have implemented various restrictions such as national lockdown orders with the closure of many businesses, schools, and public places, among other severe restrictions. Indirect effects led to deaths and a wide range of morbidity resulting from the lack of preventive care, delays in the diagnosis of disease, or interruptions in treatment of chronic conditions. The COVID-19 pandemic has indirectly led to a dramatic crisis in health systems around the world due to reorganization by allocating large proportions of health budgets to the management of COVID-19 and not to the treatment of chronic conditions as in the pre-pandemic. Additionally, the social isolation measures put in place have led to job and income losses, changes in human behavior including being kept indoors and being physically inactive due to fear of contracting the virus [4]. Furthermore, the indirect effects of the COVID-19 pandemic at the population level as a consequence of the reorganization of health care systems have been noticeable, even in countries with well-resourced and low community transmission rates. A report from Singapore shows that even though the total volume of emergency medical service calls remained constant, there was a worrying drop in the rate of achieving pre-hospital return of spontaneous circulation, even though the number of out-of-hospital cardiac arrests remained about the same [5]. The global lockdown launched by various countries around the world from March 2020, following the announcement of COVID-19 as a pandemic by the WHO, was initiated to stop the spread of the virus and to “flatten the curve” of the pandemic. In Romania, a state of emergency was instituted on 16 March 2020 for two months until 14 May 2020. However, the impact of the lockdown has had profound effects in different aspects of life including increasing the rates of anxiety and depression, among others [6,7].

Postpartum depression (PPD, described as “the thief who steals motherhood” by depriving women of the anticipated happiness of a newborn is a debilitating but treatable mental disorder, represents one of the most common complications of childbirth. PPD can appear at any time in the first year after birth. Most women develop PPD in the first three months postpartum [8,9]. The Diagnostic and Statistical Manual of Mental Disorders, Fifth Edition (DSM-5) classifies postpartum depression as a major depressive disorder with “peripartum onset”. Mothers with PPD might experience extreme sadness, decreased pleasure, low energy, anxiety, irritability, and thoughts of death. Severe cases of PPD can escalate to maternal suicide. The prevalence of postpartum depression varies between 6.5% and up to 19% [10,11].

Suicide was identified as the leading cause of maternal deaths in a Confidential Enquiry into Maternal Deaths (CEMD) report, with a rate of 10% of maternal deaths reported. However, the rate of suicide during pregnancy and in the two years following birth is lower than in men, and is falling at a faster rate. In addition, the suicide rate has been reported at two per 100,000 maternal deaths compared to 3.4 per 100,000 among all women [12]. It is considered that the highest suicide risk occurs at 9–12 months postpartum, a period when women are particularly vulnerable [13,14].

However, the postpartum period was definitely a period of increased risk for developing a major depressive episode. However, the postpartum period was clearly a period of increased risk for developing a major depressive episode. Moreover, even though there are lower incidences of depression during pregnancy in particular population groups, it remains undiagnosed particularly among racial and ethnic minorities [15]. During a period of crisis, for example, the COVID-19 pandemic, pregnancy can act as a protective factor against symptoms of depression. This can be attributed to the fact that pregnant women reported more perceived support from their partners than non-pregnant women during the pandemic. In times of crisis, partner support is a protective factor against postnatal depression, with partner support significantly predicting lower levels of depressive symptoms among pregnant women [16].

The aim of this study was threefold: to determine the prevalence of postpartum depression, to compare the prevalence of postpartum depression at two different times during the COVID-19 pandemic, and to assess the likelihood of women who gave birth during wave 4 developing postnatal depression compared to women who gave birth during wave 1 of the COVID-19 pandemic, in order to examine a possible association between the duration of the pandemic or severe measures to prevent the spread of the virus in hospitals and postnatal depression.

## 2. Materials and Methods

### 2.1. Study Design

A cross-sectional study was conducted on women who gave birth at the Obstetrics and Gynecology Clinic of the Timisoara Municipal Hospital, Romania during two different periods of the COVID-19 pandemic. Participants were interviewed using the Romanian translated version of the Edinburgh Postnatal Depression Scale (EPDS).

### 2.2. Settings

Participants were interviewed immediately after birth (days 2–4), in two different periods: 16 March 2020–31 March 2020 (during the first lockdown in Romania) and between 1 October 2021 and 14 October 2021. During the March period from 16 to 31 March 2020 when the lockdown was implemented in Romania, the anti-COVID prevention measures in the Timisoara Municipal Clinical Hospital were extremely strict (compared to the measures applied during 1–14 October 2021). In accordance with the reorganization of the public health system imposed by the Romanian Ministry of Health, the Obstetrics and Gynecology Department of the Timisoara Municipal Emergency Hospital was assigned to admit only non-COVID patients. Thus, all patients were admitted only if they had a negative PCR test performed within the last 72 h or after a PCR test on admission. Until the test results came back, the patients were isolated and if the test result was positive, the women were transferred to another hospital designed for COVID positive pregnant women. These measures, plus hospital quarantine so that husbands or other female family members did not have access to the department, were implemented in both study periods. In addition, during the period 16 March 2020–31 March 2020, women were completely isolated, with restricted contact with their newborns at birth and throughout the period of admission. Thus, mothers were not allowed to see their babies until discharge and were forbidden to breastfeed or to visit the neonatal department.

### 2.3. Participants

The study involved women who gave birth during the two periods in the Obstetrics and Gynecology Department of the Timisoara Municipal Emergency Hospital. They were aged between 18 and 45 and able to give written consent. Mothers with a known diagnosis of depression or psychotic disorders were excluded, as were women who were unable to answer the questionnaire on days 2–4 postpartum.

### 2.4. Study Size

A simple random sampling technique without replacement was used to select the participants. There have been no previous studies among populations similar to that in this study that have reported the prevalence of PPD. Thus, this study adopts the largest meta-analysis approach, which estimates the prevalence of PPD to be approximately 18%. Based on the PPD prevalence of 18%, the estimated sample size was calculated and the minimum required sample size was 79 (16–31 March 2020) and 64 (1–14 October 2021), respectively, at the 95% confidence interval, 5% margin of error, and 80% power. This number was increased to 95 and 77, respectively, to overcome 20% non-response. In the two study periods, the number of women giving birth was 120 between 16 and 30 March 2020 and 88 between 1 and 14 October 2021. Between 16 and 31 March 2020, of the 95 women assessed for eligibility, four (4.21%) refused to participate and three (3.15%) women were unable to answer the questionnaire. Between 1 and 14 October 2021, of the 77 women assessed for eligibility, eight (10.3%) refused to participate and three (3.89%) woman were unable to answer the questionnaire.

### 2.5. Edinburgh Postnatal Depression Scale

Symptoms of depression were screened using the Edinburgh Postnatal Depression Survey (EPDS). This is a 10-item self-assessment questionnaire with answers that are scored from 0 to 3 and has a maximum score of 30 [17]. A cutoff score of 13 had a sensitivity of 0.66 and a specificity of 0.95 in identifying women with clinical symptoms of postpartum major depression, while a cutoff score of 10 had a sensitivity of 0.85 and a specificity of 0.84 [18].

### 2.6. Data Sources/Measurement

Questionnaires were administered and completed face-to-face, with a researcher assisting women who failed to complete the questionnaires. Along with the questionnaire to determine postpartum depression, participants were administered another questionnaire that included questions about socio-demographic status (age, place of residence, educational status, occupation), pregnancy (complications during pregnancy), childbirth (gestational age at birth, type of delivery), and newborn (birth weight, fetal gender, Apgar score at 1 min). A Microsoft Office Excel database was created to record the results.

### 2.7. Outcomes

Outcomes of interest were the prevalence of postpartum depression symptoms during the two periods of the COVID-19 pandemic and the risk of postpartum depression in relation to in-hospital COVID-19 prevention measures and the pandemic period.

### 2.8. Statistical Methods

The statistical analysis was performed using RStudio. For descriptive statistics, continuous variables were expressed as mean ± standard deviation or median and categorical variables as proportions. Comparison of continuous variables was performed using the t-test for independent samples or Mann–Whitney U test, and comparison of categorical variables was performed using the χ2 test. Univariate or multivariate binary logistic regression was used to examine the relationship between the independent variables and the dependent variable (EPDS score > 13). *p* < 0.05 was considered significant. Place of residence (urban vs. rural), educational status (low/middle vs. high school/higher education), mother’s occupation (working vs. not working), type of birth (natural vs. cesarean), presence of at least one pregnancy complication (such as pre-eclampsia, intrauterine growth restriction, gestational diabetes, preterm birth, etc.), maternal age, and Apgar score (less than or equal to 7) were used as confounders in the regression models. The subscales anhedonia, anxiety, and depression were also used as confounders.

## 3. Results

### 3.1. Sample Descriptive Statistics

Participant characteristics are presented in Table 1. Mean participant age was 29.06 years old. The majority of participants were from rural areas, had a high level of education (high school or higher education), and were primiparous. Additionally, most of the women included gave birth by caesarean section, with a mean birth weight of the newborn of 3133 g. The characteristics of the participants were similar between the two periods apart from the place of origin and educational status.

### 3.2. Prevalence of Postpartum Depression Symptoms and Comparation between Two COVID-19 Pandemic Waves

**Figure 1:**
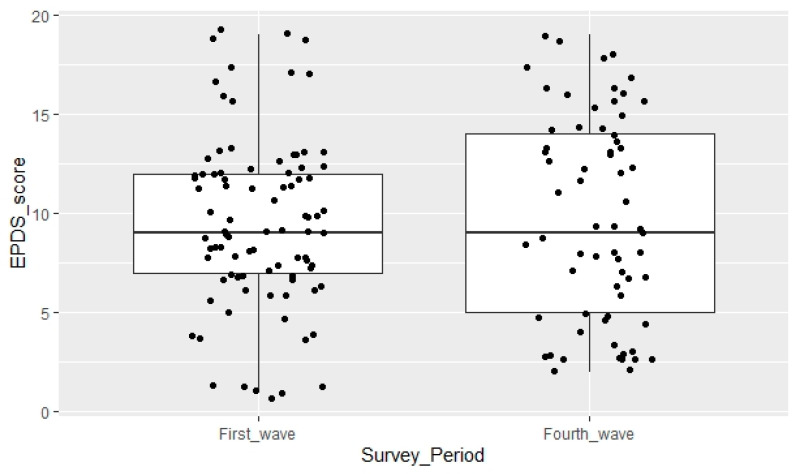
Mean EPDS score among the two survey groups: first wave of the COVID-19 pandemic in Romania (16 March 2020–31 March 2020) and during the fourth wave of the COVID-19 pandemic in Romania (1 October–14 October 2021), respectively.

Among the 154 participants the mean EPDS score was 9.66. Figure 1 illustrates the mean EPDS score in the two survey groups, no statistically significant difference was observed between them (*p* = 0.73).

The overall prevalence of postpartum depression (EPDS score > 13) was 18.8%, with a statistically significantly higher rate among participants surveyed between 1 October 2021 and 14 October 2021 (during the fourth wave of the COVID-19 pandemic in Romania) (Table 2).

### 3.3. Association of Participant Characteristics and Survey Period during the COVID-19 Pandemic with Postpartum Depression

Univariate analysis showed that births between 1 and 14 October 2021 in wave four of the COVID-19 pandemic in Romania (survey period) and the participants’ occupation were independent risk factors for postpartum depression (Table 3).

After adjusting for confounding factors, the multivariable logistic regression models showed a significant association between birth between 1 October and 14 October 2021, which was wave four of the COVID-19 pandemic in Romania, and the risk of postpartum depression (EPDS score of 13) (Table 4).

## 4. Discussion

Postpartum depression is the most common psychiatric disorder after childbirth and is included in the Diagnostic and Statistical Manual of Mental Disorders of the American Psychiatric Association as a major depressive episode “with peripartum onset if the onset of mood symptoms occurs during pregnancy or within four weeks of delivery”. However, this condition is poorly studied and often undiagnosed [19,20,21].

The EPDS is a 10-item scale that was developed as a unidimensional screening tool for postpartum depression, being useful even in immediate postpartum use [17,22]. In addition, a positive correlation was reported between immediate postpartum EPDS scores and scores on days 30–40 [23]. Moreover, various studies have reported that the EPDS identifies different dimensions of postpartum mental health, mainly anhedonia, anxiety, and depression [24,25,26,27,28].

In the present study, we assessed women immediately after childbirth for postpartum depression using EPDS questionnaires during two waves (Wave 1 and Wave 4) of the COVID-19 pandemic.

The results showed that the overall prevalence of PPD was 18.8%, similar to the results of the largest meta-analysis reported up to the present where the prevalence of PPD was found to be 17.22% [10]. However, some studies in early postpartum women showed a prevalence of depressive symptoms (EPDS > 13) of 13.0%, comparable to the prevalence among women in our study who gave birth during the first wave of the COVID-19 pandemic [29].

It was found that women who gave birth during wave 4 of the pandemic had a significantly higher rate of PPD compared to those who gave birth during wave 1 of the pandemic (28.8% vs. 11.4%; *p* = 0.006). In addition, the univariate logistic regression showed that women who gave birth during wave 4 of the COVID-19 pandemic were 3.15 times more likely to develop postpartum depression than women who gave birth during wave 1 of the COVID-19 pandemic. It should be noted that these results come in the context that women who gave birth during wave 1 were subjected to more stringent in-hospital measures to control transmission of the virus, in particular, complete isolation of the newborn and a breastfeeding restriction until discharge. In a study conducted before the COVID-19 pandemic and during the first wave of COVID-19 infections, Pariente et al. demonstrated that women who gave birth during the first wave of COVID-19 pandemic had a lower risk of depression compared to the comparison group of women who did not give birth during the pandemic [30]. These results may be explained by the presence of the most stringent quarantine restrictions during the first pandemic wave, whereby pregnant women and their families stayed at home together and were able to obtain more support from their loved ones. However, several studies have reported that lockdowns and social distancing during the pandemic had a significant role in affecting women’s mental health in the postpartum period [31,32,33,34].

The PPD prevalence of 28.8% found among women who gave birth during wave 4 of the COVID-19 pandemic was similar to the 27.43% reported in other studies that also showed PPD rates during the pandemic [35]. The COVID-19 pandemic is a significant global stress event. Social isolation and quarantine are efficient measures to prevent the spread of viral transmission, but have negative effects on mental health, especially in the postpartum period, when women are at risk [36]. It is also believed that reduced physical activity during pregnancy, feelings of isolation, job loss, fear of visiting hospitals during the pandemic, lack of a support person during childbirth, changes in the birth plan, fear of not being able to visit family after childbirth, or lack of childcare facilities are factors that have contributed to increased depressive symptoms among women in the postpartum period [37].

Health workers should encourage new mothers to discuss their mental state as well as seek help for any symptoms of depression. It should be noted that during the pandemic, mental health screenings need to be tailored. Thus, remote health services play an important role during this period [37]. It is considered that improving access to mental health services for the population during this period should focus on telemental health or telemedicine services. In addition, the creation of social support networks or online support groups could also help during this pandemic. However, rates of use of telemedicine services remain low due to several factors such as legal issues or patient and doctor non-familiarity [38].

Of the social-demographic factors, the type of employment status of women had an effect on PPD, more specifically, women who worked had a higher risk of developing PPD than those who did not work. Guvenc et.al also found that working women had a higher risk of postpartum depression [39]. However, contrary to these results, another study showed that nonworking women had a higher risk of developing PPD [40]. Future studies could show the effect that the pandemic has had on working mothers.

We found no significance for characteristics such as age, education level, place of residence and type of birth, number of children and sex of the infant, or pregnancy complications. In contrast, Houston et al. described an association between strength of preference for vaginal delivery, delivery mode undergone, and postpartum depression [41].

Regarding the type of birth, the high rate of caesarean sections among the women in this study is noteworthy. It should be noted that an increased rate of caesarean section has been observed at the national level, which has been increasing in recent years, unrelated to the COVID-19 pandemic. In 2012, the rate of caesarean sections at the national level was 41.2% of all births, and in 2017, Romania was ranked second in Europe after Cyprus regarding this rate [42,43]. This high rate is mainly due to the desire of women, most caesarean sections being on request without an obstetric indication. In addition, many doctors recommend caesarean section without a strong medical indication, most likely due to fear of malpractice [42]. The increased rate of cesarean section in our study, even above the national average, may be due to the fact that our hospital is a tertiary center, where many cases with an indication for cesarean section are transferred from smaller centers where it is not possible to perform such procedures. Contrary to the results observed in our study, where the type of birth is not associated with the likelihood of depressive symptoms, some studies suggest that cesarean section increases the risk of PPD [44].

This study has several limitations. First, being a cross-sectional study, it did not allow clear causal relationship between PPD and factors associated with it. Second, the short time between birth and questionnaire completion may not allow us to observe the long-term impact on mental health. Moreover, participants may have provided answers that they consider socially acceptable.

## 5. Conclusions

During the COVID-19 pandemic, the overall prevalence of PPD was similar to that before the pandemic. However, there was a marked increase in the PPD rate among women who gave birth during wave 4 of the pandemic. These findings indicate that the pandemic has a major impact on the occurrence of depressive symptoms after childbirth and can be used to formulate psychological interventions to minimize depression among these women. In addition, the low PPD rate during wave 1 may prove that in-hospital restrictions to prevent COVID-19 have no impact on depressive symptoms among postpartum women.

## Figures and Tables

**Table 1:** The characteristics of participants evaluated between 14 March 2020 and 31 March 2020, and between 1 October2021 and 14 October 2021.

Characteristics	Total (*n* = 154)	Survey Period		*p*-Value
		16 March 2020–31 March 2020 (*n* = 88)	1 October–14 October 2021 (*n* = 66)	
Age (Mean + SD)	28.06 ± 5.71	27.5 ± 5.92	28.8 ± 5.38	0.15
<25 years	48/31.2%	30/34.1%	18/27.3%	0.36
>25 years	106/68.8%	58/65.9%	48/72.7%	
Place of residence (count/percentage)				
Rural	80/51.9%	38/43.2%	42/63.6%	0.01
Urban	74/48.1%	50/56.8%	24/36.4%	
Educational Status				
Low/Middle Education ^1^	38/24.7%	32/36.4%	6/9.1%	<0.01
Highschool/Higher Education	116/75.3%	56/63.6%	60/90.9%	
Occupation (count/percentage)				
Not Working	58/37.7%	33/40.9%	22/33.3%	0.33
Working	96/62.3%	52/59.1%	44/66.7%	
Parity (count/percentage)				
Primiparous	84/54.5%	40/45.5%	48/66.7%	0.09
Multiparous	70/45.5%	48/54.5%	22/33.3%	
Complications during pregnancy (count/percentage)	66/42.9%	38/43.2%	28/42.4%	0.92
Gestational age at birth (Mean + SD)	38.18 ± 2.21	38.0 ± 2.11	38.4 ± 2.35	0.31
Type of delivery (count/percentage)				
Vaginal delivery	44/28.6%	28/31.8%	16/24.2%	0.30
Cesarean Section	110/71.4%	60/68.2%	50/75.8%	
Birth Weight in grams (Mean + SD)	3133 ± 576	3078 ± 520	3206 ± 639	0.17
Fetal gender (count/percentage)				
Male	90/58.4%	42/47.7%	48/72.7%	0.02
Female	64/41.6%	46/52.3%	18/27.3%	
Apgar score (Median)	9	9	9	0.25

^1^ Low/middle education includes illiterate/no education, elementary school, and secondary education; SD = standard deviation.

**Table 2:** Description of EPDS score results among the 154 participants surveyed in the two periods.

Variable	Total	Survey Period		*p* Value
		16 March 2020–31 March 2020	1 October–14 October 2021	
EPDS score (mean ± SD)	9.66 ± 4.65	9.55 ± 4.30	9.80 ± 5.09	0.73
Total EPDS score > 13	29/154 (18.8%)	10/88 (11.4%)	19/66 (28.8%)	0.006
Total EPDS score > 10	67/154 (43.5%)	36/88 (40.9%)	31/66 (47.0%)	0.45
Anhedonia subscale (mean ± SD)	1.27 ± 1.48	1.34 ± 1.65	1.17 ± 1.22	0.47
Anxiety subscale (mean ± SD)	5.79 ± 2.74	5.64 ± 2.56	6.0 ± 2.97	0.41
Depression subscale (mean ± SD)	2.62 ± 2.21	2.57 ± 2.33	2.70 ± 2.06	0.72

**Table 3:** Univariate logistic regression for the association between participants’ characteristics, childbirth period, and postpartum depression (EPDS score ≥ 13) during the COVID-19 pandemic.

Variables	Estimate	Std. Error	Statistic	*p*-Value	95% CI	
					Lower	Higher
Survey Period	3.15	0.43	2.66	0.01	1.38	7.61
Place of Residence	1.01	0.41	0.03	0.98	0.45	2.28
Education Status	2.34	0.57	1.48	0.14	0.83	8.36
Occupation	2.73	0.49	2.04	0.04	1.10	7.81
Type of Delivery	0.59	0.43	−1.23	0.22	0.25	1.40
Pregnancy Complication	0.93	0.42	−0.18	0.86	0.40	2.09
Age	1.20	0.44	0.43	0.67	0.50	2.79

Note: Survey period was for pandemic wave 1 (16 March 2020–31 March 2020) compared with pandemic wave 4 (1–14 October 2021).

**Table 4:** Multivariable logistic regression models for the association between the COVID-19 pandemic period and postpartum depression.

Model	Estimate	Std. Error	Statistic	*p*-Value	95% CI	
					Lower	Higher
Survey Period * Place of Residence	3.34	0.44	2.72	0.01	1.43	8.26
Survey Period * Education Status	2.82	0.45	2.30	0.02	1.19	7.08
Survey Period * Occupation	3.04	0.44	2.54	0.01	1.31	7.41
Survey Period * Type of Delivery	3.40	0.44	2.78	0.01	1.47	8.36
Survey Period * Pregnancy Complication	3.15	0.43	2.66	0.01	1.38	7.61
Survey Period * Age	3.23	0.43	2.70	0.01	1.40	7.84
Survey Period * APGAR < 7	1.17	0.43	3.24	0.007	1.37	7.66
Survey Period * Anxiety Score	0.84	0.63	2.33	0.18	0.66	8.11
Survey Period * Anhedonia Score	1.44	0.47	4.24	0.002	1.66	10.8
Survey Period * Depression Subscale Score	1.95	0.62	7.07	0.002	2.09	23.8

Note: Survey period * was for pandemic wave 1 (16 March 2020–31 March 2020) compared with pandemic wave 4 (1–14 October 2021).

## Data Availability

The data presented in this study are available on request from the corresponding author.
